# Book Review: The History of the Basel Institute for Immunology

**DOI:** 10.3389/fimmu.2018.00566

**Published:** 2018-03-19

**Authors:** Donald F. Gerson

**Affiliations:** ^1^PnuVax Inc., Kingston, ON, Canada

**Keywords:** Basel Institute for Immunology, Roche, immunology, Nobel Prize, Niels Jerne, Georges Kohler, Susumu Tonegawa, Charley Steinberg

Prof. Ivan Lefkovits writes from the unique perspective of being present for the entire 32 years that the Basel Institute for Immunology (BII) was the global focus of immunological research and discovery. The book is the story of the place and people leading to the discovery of the genetic mechanisms of immunoglobulin diversity and the elucidation of the immunological network, leading quickly to three Nobel Prizes.

This outcome is the result of the careful curation and caretaking by Prof. Niels Jerne of the Members of the Institute: his management paradigm was unique, led directly to success, and is an important case study in effective and efficient scientific research management. Ralph Waldo Emerson said that “*A man Caesar is born, and for ages after we have a Roman Empire* … *An institution is the lengthened shadow of one man*…”^1^: Niels Jerne gave birth to the Network Theory and the BII, and for ages after we have Immunology.

The BII was established by Hoffmann-La Roche executives Adolf Jann and Alfred Pletscher, helped by Johann-Rudolf Frey, in 1968 to create new scientific knowledge of the then poorly understood immune system, for the future benefit of humanity and for the pharmaceutical industry by investigating a key physiological system that combats disease, and eventually identifying new pharmaceutical targets and therapeutics. The founders understood it was a philanthropic long-term economic development project supported exclusively by Roche, which would foster the whole Swiss pharmaceutical industry and provide meaningful local employment. Key to their prescient thinking was that while Roche provided all funding, the Institute was independent and not required to directly benefit the company. This freedom, so rare today, allowed the great creativity which followed.

Prof. Lefkovits has carefully and passionately recounted the remarkable story of BII, supporting it with photos and background data embedded in QR codes.

Niels Jerne was selected to lead BII and given the freedom to design it *de novo* both as a building and as a scientific community, without corporate constraints and hierarchies. Niels had many special attributes: He gathered detailed accurate experimental data, confirmed its veracity, theorized, and repeated the cycle. He understood how scientists worked. Both of these became the soul of BII. His early measurements of diphtheria antisera told him that antisera must contain multiple antibodies each with different binding strengths, contrary to contemporary ideas. He persisted, wanted to understand antibody differences, and proposed a comprehensive natural-selection theory for antibody diversity. This was the conceptual basis for BII.

His building design encouraged communication, a circular hallway intersected the coffee room, labs connected vertically by circular staircases made a 3D matrix where we met and interacted continuously. Once the building was designed, Prof. Lefkovits supervised its construction, and Niels searched the world for the complement of scientists who could best develop a new understanding of the immune system—a mix of biologists, physiologists, biochemists, virologists, and molecular biologists with different backgrounds and each with insatiable curiosity, with both long and short term engagements, and all focused on elucidating the immune system. They formed one of the world’s largest and yet fluid assembly of bright active individuals that self-coalesced to first imagine and then solve a multitude of immunological problems. With all these advantages, and without time-consuming grant applications and bureaucracy, everyone spontaneously maximized their personal and the group’s capabilities to achieve the Institute’s overarching goals.

*The History of the BII* gives a year-by-year account of the Members’ increasingly greater understanding of the immune system and the mastery of several exceptional individuals: Georges Kohler, Susumu Tonegawa, Charley Steinberg, and many others. Jerne’s wisdom dominated and focused the direction of BII from 1970 to his retirement in 1980. The immunological research was both idiosyncratic and wide, ensuring deep discovery of the immune system. Initially, some looked directly at immunity: Louis Du Pasquier studied frog immunity, Ivan Lefkovits studied the initiation of the immune response to antigens, Dietmar Braun studied antibody diversity, Helmut Pohlit studied tolerance, and Ethel Jacobson studied immune suppression. Others investigated biochemical and cellular immune processes: Theo Staehelin and Max Schreier focused on protein synthesis, Georges Roelants and Brigitte Askonis studied lymphocyte differentiation, Ruggero Ceppellini and coworkers studied HLA, Seung-Il Shin studied lymphocyte fusion, and Stephen Fazekas investigated influenza virus antigens. Over the years, 500 individuals gave a wide-angle multidimensional view of the immune system through multiple synergies leading to more discoveries. These diverse seeds led to today’s highly detailed and very useful comprehension of the immunological ecosystem.

Basel Institute for Immunology was a major ganglion in the international immunological community when handwritten letters dominated communication and the arrival of a visiting scientist brought otherwise unobtainable knowledge. The constant flux of visitors thorough BII hybridized thoughts and stimulated research. Some were musicians and artists, and the intersection of science and art remained a key BII theme—scientists wrote and presented multiple musical and theatrical performances celebrating the latest immunological findings.

A question often asked today was raised at the recent launch of this book in Basel: what was the return on Roche’s investment? The answer is clearly the current plethora of therapeutic protein biopharmaceuticals: antibodies, immune modulators, and hybrids; Genentech’s acquisition; and many new companies utilizing immunological phenomena first elucidated at BII. The few millions spent on BII are less than the cost of developing one major new product today, have yielded many billions in pharmaceutical revenues, and will continue to do so for many decades to come. Management change and financial constraints closed the BII and ended this rich bounty.

This story is carefully presented and beautifully illustrated in *The History of the Basel Institute for Immunology*, a history of a very special time, place, and people, which is a lesson for the future on how to structure a scientific revolution. The usual dictum is that if we do not learn from history we are destined to repeat it; in this book, we have the opportunity to learn from the history of an incredible institution so we can repeat it, each in our own way.

## Author Contributions

The author confirms being the sole contributor of this work and approved it for publication.

## Conflict of Interest Statement

Author DFG was employed by company PnuVax Inc. Authors declare no competing interests.

## Footnotes

^1^Self-Reliance, Ralph Waldo Emerson, 1841.

